# Outpatient geriatric health care in the German federal state of Mecklenburg-Western Pomerania: a population-based spatial analysis of claims data

**DOI:** 10.1186/s12913-024-10888-2

**Published:** 2024-04-12

**Authors:** Nils Pfeuffer, Franziska Radicke, Maren Leiz, Kilson Moon, Wolfgang Hoffmann, Neeltje van den Berg

**Affiliations:** 1 Section Epidemiology of Health Care and Community Health, Institute for Community Medicine, University Medicine Greifswald, Ellernholzstr. 1-2, 17489 Greifswald, Germany; 2 Jade University of Applied Science, Ofener Straße 16, 26121 Oldenburg, Germany

**Keywords:** Claims data, Spatial analysis, Rural healthcare, Geriatrics, Geriatric assessment, Utilization of geriatric care

## Abstract

**Background:**

Due to unidentified geriatric needs, elderly patients have a higher risk for developing chronic conditions and acute medical complications. Early geriatric screenings and assessments help to identify geriatric needs. Holistic and coordinated therapeutic approaches addressing those needs maintain the independence of elderly patients and avoid adverse effects. General practitioners are important for the timely identification of geriatric needs. The aims of this study are to examine the spatial distribution of the utilization of outpatient geriatric services in the very rural Federal State of Mecklenburg-Western Pomerania in the Northeast of Germany and to identify regional disparities.

**Methods:**

Geographical analysis and cartographic visualization of the spatial distribution of outpatient geriatric services of patients who are eligible to receive basic geriatric care (BGC) or specialized geriatric care (SGC) were carried out. Claims data of the Association of Statutory Health Insurance Physicians in Mecklenburg-Western Pomerania were analysed on the level of postcode areas for the quarter periods between 01/2014 and 04/2017. A Moran’s I analysis was carried out to identify clusters of utilization rates.

**Results:**

Of all patients who were eligible for BGC in 2017, 58.3% (*n* = 129,283/221,654) received at least one BCG service. 77.2% (*n* = 73,442/95,171) of the patients who were eligible for SGC, received any geriatric service (BGC or SGC). 0.4% (*n* = 414/95,171) of the patients eligible for SGC, received SGC services. Among the postcode areas in the study region, the proportion of patients who received a basic geriatric assessment ranged from 3.4 to 86.7%. Several regions with statistically significant Clusters of utilization rates were identified.

**Conclusions:**

The widely varying utilization rates and the local segregation of high and low rates indicate that the provision of outpatient geriatric care may depend to a large extent on local structures (e.g., multiprofessional, integrated networks or innovative projects or initiatives). The great overall variation in the provision of BGC services implicates that the identification of geriatric needs in GPs’ practices should be more standardized. In order to reduce regional disparities in the provision of BGC and SGC services, innovative solutions and a promotion of specialized geriatric networks or healthcare providers are necessary.

**Supplementary Information:**

The online version contains supplementary material available at 10.1186/s12913-024-10888-2.

## Background

Demographic change is a public health challenge worldwide [1]. It is internationally observed that access to quality health care for elderly people in rural areas is worse compared to urban regions [2, 3]. The demographic change is often accentuated in rural regions. Concurrently, the establishment of specialized as well as basic health care facilities in rural areas is often less attractive for healthcare providers for a number of reasons, e.g., economic issues or working conditions. In addition, great distances in rural areas can be a great barrier for less mobile elderly people, especially where the availability of public transport is limited [2, 4–6]. Studies on suboptimal prescribing and adverse drug reactions in elderly patients show the importance of access to specialized geriatric in- and outpatient care [7, 8].

According to the German Association for Geriatric Medicine, geriatric patients are often defined by advanced age and ≥ 2 geriatric-typical syndromes at the same time [9]. Geriatric-typical syndromes are for example frailty, decubitus and a tendency to fall [9, 10].

Geriatric patients have a higher risk for multi-morbidity, chronic conditions and functional decline. They are often frail or pre-frail, which means an increased vulnerability that is associated with adverse health outcomes [11] and general weakness, poor endurance, weight loss and/or undernourishment, low level of activity and unsteady gait [12]. A cross-sectional study, based on representative data from the “German Health Interview and Examination Survey for Adults” of the Robert-Koch-Institute conducted between 2008 and 2011, estimates the average frailty and pre-frailty prevalence in geriatric patients at 41.4% in Germany [13]. Since the prevalence was only determined for the 65 to 79 years old persons and no home visits were carried out to examine patients with limited mobility, the prevalence is likely to be underestimated [14].

Moreover, cognitive decline is common in old age and is associated with an increased number of adverse medical events. Cognitively impaired geriatric patients have a higher risk of poor functional recovery during rehabilitation ward [15], loss of independence after discharge from acute care, and mortality [16]. The socio-economic burden is also increased in geriatric patients with cognitive impairment due to a higher demand for formal [17] and informal care [18].

An effective treatment of geriatric patients’ needs to focus on the specific individual needs, interactions among conditions or treatments, the patient´s individual preferences, beliefs, goals, prognosis, and the multifactorial nature of geriatric morbidity [19]. In this context, a geriatric assessment serves as an instrument for a comprehensive examination of a geriatric patient’s health situation and individual resources. It is a basis for comprehensive care that focuses on preserving the patients’ independence and autonomy. This is in line with the national quality assurance guideline on the specialized geriatric diagnostic by the national Association of the Statutory Health Insurance Physicians (ASHIP) that defines the requirements of specialized geriatric practices in terms of the necessary qualifications and resources of the healthcare providers in Germany. It also determines the form and content of the specialized geriatric diagnostic in Germany [20]. Several studies on the effects of a comprehensive geriatric assessment and consecutive treatment have shown its efficacy in terms of reducing functional decline, improving mental health [21], decreasing risk of nursing home placement [22, 23], and delaying the progression of frailty [24, 25]. However, especially for rural living older adults it is difficult to access specialized geriatric healthcare due to longer distances between patients and healthcare providers. Outpatient geriatric practices can help to improve the access of older rural living adults to geriatric healthcare [26].

In Germany, the provision of geriatric care is heterogeneous, because structures and qualifications vary between the federal states. Basically, general practitioners (GPs) are responsible for coordinating diagnostic measures and treatment of geriatric patients. With an additional special training, GPs can receive an approved qualification in geriatrics. In four (of 16) federal states, physicians can further train to become a specialist in geriatrics [27, 28]. However, especially in outpatient geriatric care, the number of GPs with any geriatric qualification is low. Until 2015, less than 1% of the GPs had an approved qualification in geriatric care. And until the second half of 2016, the additional effort that the specialized practitioners (SP) needed to invest in comprehensive care of geriatric patients were not adequately financed by the German reimbursement schemes [29].

this study aims to investigate the utilization of outpatient geriatric health care in a rural region, the Federal State of Mecklenburg-Western Pomerania (MWP) in the northeast of Germany. The analysis includes the spatial distribution of the utilization of the outpatient services for basic geriatric care (BGC) and specialized geriatric care (SGC). The primary research question is whether there are regional disparities in the utilization of BGC and SGC services in Mecklenburg-Western Pomerania (MWP). Secondary research questions are: (1) can spatial patterns of utilization be identified, and (2) if regional differences can be identified, are distance or care provider density possible explanations for these differences?

## Methods

### Design and data

This study is a spatial analysis of the distribution of the utilization of outpatient geriatric healthcare services in MWP. The analysis is based on claims data from the Association of Statutory Health Insurance Physicians in MWP (ASHIP-MWP). The statutory health insurance physicians are collecting the ASHIP-MWP data for the reimbursement of their services which they are providing for patients insured by a Statutory Health Insurances (SHI). In 2020, about 87% of the inhabitants of Germany were members of a SHI [30]. The data contained the kind of reimbursed services, reimbursement quarter and date, pseudonymized practice identification number (practice ID), practice location (postcode), patient anonym, patient residence (postcode), patient date of birth, and sex. The data covers the years 2014–2017. Some geriatric medical services were new in the reimbursement schedule and became reimbursable from the 3rd quarter of 2016. That is why the year 2017 is the data basis for most of the analyses. The total number of all geriatric services per quarter was calculated for all four calendar years in order to analyse the development of utilization over time.

The reimbursement catalogue of the SHI accredited physicians (abbreviated in German as EBM) defines 2 groups of patients that are eligible to receive geriatric services:

1. For BGC: the patient has to be at least 70 years old and has to have at least one geriatric syndrome (according to Table 1) or a recognized care level according to the statutory long-term insurance or, age-independent, the patient is diagnosed with F00-F02, G30 or a G20 diagnosis.
2. For SGC, the patient has to be at least 70 years old and has to have at least two geriatric syndromes (see Table 1) or one geriatric syndrome and a recognized care level according to the statutory long-term insurance [31].

**Table 1:** Geriatric syndromes according to the EBM of the national ASHIP

Geriatric syndromes
Multifactorial mobility disorder including tendency to fall and dizziness
Cognitive, emotional or behaviour-related complex impairments
Frailty-Syndrome (combination of unintended weight loss, physical and/or cognitive fatigue, muscular insufficiency, reduced gait velocity, and reduced physical activity)
Dysphagia
Incontinence
Therapy-refractory chronic pain syndrome

The diagnostic and therapeutic services of the BGC and SGC are comprised as follows:

- BGC includes a basic geriatric assessment (BGA) and a basic geriatric treatment (BGT). The BGA can be provided up to twice a year. Beyond the assessment and/or monitoring of motor, emotional and cognitive functional impairments, it includes an obligatory examination of self-care abilities using standardized assessment instruments. Other services are optional, e.g., an assessment of cognitive limitations or recommendations on adaptions of the home environment to existing individual disabilities. Thereafter, basic geriatric treatment (BGT) is carried out by the GP who, based on the geriatric assessment, coordinates, implements and performs therapeutic measures as well as monitors and manages the medications of the patients. Both services are conducted by the GP. An approved geriatric qualification is not necessary to be allowed providing these services. The BGT can be accounted up to four times per year per patient by the GP.
- SGC requires a cooperation between the GP and a SP with an approved geriatric qualification or a geriatrician. If both sides identify a need for further geriatric treatment, a comprehensive geriatric assessment (CGA) can be carried out by the geriatric SP or the geriatrician. In contrast to the BGA the CGA is a more extensive standardized assessment of the patient’s social and health situation. Following the CGA, the GP or the SP and the geriatrician, respectively are allowed rolling out a comprehensive and team-based treatment (CGT) including, e.g., ergo-, physio-, or speech therapy. The treatment bases on specific geriatric treatment goals, and a detailed treatment plan [31].

An overview about the main information on the BGC and SGC in Germany is given by the synoptic Table A1 in the multimedia appendix. Figure A2 of the multimedia appendix displays an overview about the patient pathways of geriatric patients in outpatient care according to the German reimbursement schemes. It mentions also the reimbursement codes for the corresponding services of BGC and SGC, and who is allowed to account for one or the other. The ASHIP-MWP provided pre-selected data by applying the geriatric definitions according to the EBM (see Table 1) and having used the following diagnosis as inclusion criteria (see Table 2).

**Table 2:** Diagnoses that has been used as inclusion criteria (in addition to the age of the patients) for defining the eligibility

Diagnosis title	ICD 10 Code
Entitled for a nursing care service	274.0–274.3
Multifactorial mobility disorder including tendency to fall and dizziness	R42, R29.6
Cognitive, emotional or behaviour-related complex impairments	F03, F04, F05.0 - F05.9, R41.8, R46.4
Frailty-Syndrome	R54, R68.8
Dysphagia	R13
Incontinence	R32
therapy-refractory chronic pain syndrome	R52.1; R52.2
Immobility	R26.3
Decubitus ulcers	L89
Malnutrition	R64, E41, E46
Disorders in the fluid and electrolyte balance	E87.8
Sleep disorders	G47
Depression, anxiety disorder	F32.9, F41.9
Sensory disturbance	R20.8
Severe visual and hearing impairment	H91.1, H52.4
Dementia	F00-F02
Alzheimer disease	G30
Primary Parkinson syndrome	G20

Patients who are eligible to receive SGC were also included in the group of beneficiaries for BGC.

### Population

The number of inhabitants of MWP in 5-year age groups at the municipal level was derived from the Central Information Register of MWP on January 18, 2018. The municipalities were linked to the respective postcode areas, so that in the next step the population data could also be linked to the postcode areas. Additional population data required for age standardization was obtained from the statistical state office of MWP (for the year 2017).

### Statistical and spatial analysis

The geriatric services were descriptively analysed. For the BGC and SGC services, the proportion of patients treated in relation to all eligible patients (entitled for geriatric care per category) was calculated.

To prepare the data for spatial analysis and visualization, the locations of the GPs in MWP (*n* = 742), were geocoded on the basis of address data from the ASHIP-MWP from 2015. The density of GPs was calculated in relation to the total population and to the population aged ≥ 70 years. A possible association between the density of GPs and the number of treated patients was examined both with a linear regression (dependent variable: number of treated patients; independent variable: density of doctors) and with a spatial analysis.

Spatial autocorrelation (global and local Moran’s I) was carried out to examine whether the pattern of the utilisation rates of the geriatric services were random, dispersed, or clustered over the postcode areas. Since Moran’s I analysis bases on neighbourhood relationships, one postcode area without direct neighbours (the island Hiddensee) was excluded from the analysis, as an inclusion could have biased the analysis. Patients who received BGC or SGC services in MWP but lived outside of MWP (*n* = 8,472) were excluded from the spatial analyses.

Sensitivity analyses on the basis of the total population and the number of GPs were carried out. With a geographical information system, the number of inhabitants per place of residence was aggregated by postcode so that they could be related to the number of GPs in the respective postcode areas and to the claims data. To calculate the GP-density, the geocoded locations of the GP practices were aggregated by postcode area. After that, the GP-density per 10,000 inhabitants of the total population and of the population aged 70 years or older at postcode level was calculated. The results were cartographically visualized.

The utilization rates within the postcode areas were age-standardized with the population of MWP as of December 31, 2017. Statistical and inhabitant related calculations were carried out with Stata® Statistical Software, Release 14.1, StataCorp 2015.

The results of the spatial analyses were cartographically visualized with the Geographic Information System ESRI®ArcGIS™ 10.7.1 Esri Inc., Redlands/California (USA).

## Results

### Basic geriatric outpatient services

221,654 patients (75.3% of all inhabitants of Mecklenburg-Western Pomerania aged ≥ 70 years) were eligible for BGC services in 2017 according to the criteria of ASHIP-MWP and had at least one doctor’s visit in 2017. Thereof, 58.3% (*n* = 129,283) actually received at least one geriatric service in 2017.

Between 2014 and 2017, the number of patients with at least one basic geriatric assessment (BGA) per year stayed at about the same level. The number of patients with at least one basic geriatric treatment (BGT) per year increased slightly in this period (see Table 3).

**Table 3:** Number of patients with at least one basic geriatric care service per year

Year	Number of patients with basic geriatric assessments (N)	Number of patients with geriatric treatment (N)
2014	110,697	108,797
2015	113,891	116,637
2016	113,734	118,399
2017	113,581	118,755

### Specialized geriatric outpatient services

*N* = 95,171 patients (32.3% of the population of Mecklenburg-Western Pomerania ≥ 70 years) were entitled to SGC in 2017 according to the criteria of the ASHIP-MWP. Thereof, 77.2% (*n* = 73,442) received at least one geriatric service (including BGC and SGC services). However, only 0.4% (*n* = 414 patients) of this group actually received at least one SGC service.

Of these 414 patients, *n* = 303 times the GP accounted for a consultation with a SP to assess the need for a CGA and *n* = 270 times the SP accounted for the preliminary clarification of the CGA. Of these, 173 times the GP and the SP accounted for the consultation with each other. For a majority of patients, 97.1% (*n* = 168), both services were accounted by one and the same practice and, of these, *n* = 158 (91.3%) times both parts were accounted at the same day. The CGA itself was received by 129 patients. For *N* = 14 of these patients, a prior cooperation, which is actually mandatory for the CGA, was not documented. This may be due, for example, to a consultation in the previous year (2016) or otherwise incomplete data. Only 55 patients received all three SGC services, including the consultation of GP and SP in the forefield, and the CGA itself. 98.2% (*n* = 54) of these patients received all three services in the same practice. The CGT was utilized by *n* = 89 patients in 2017. 46% (*n* = 41) of them received all 4 SGC services, and all of them (100%) received these services at the same practice.

Table 4 shows the number of patients who received at least one SGC service for the last two quarters of 2016 and during the year 2017.

**Table 4:** Number of patients with specialized geriatric services

Year	GP Part of Cooperation	SP Part of Cooperation	Comprehensive Geriatric Assessment	Comprehensive Geriatric Treatment
2016*	383	157	54	115
2017	303	270	129	89
Total	686	427	183	204

* The services are only offered since the second half of 2016

In contrast to BGC, patients utilized SGC services in most cases only once per year during the observation period.

### Spatial analysis

In 2017, geriatric services were provided in GPs’ practices with 890 different practice ID in Mecklenburg-Western Pomerania. These GP-practices were located in 179 different postcode areas. From 2014 to 2017, both the number of practices and the number of postcode areas decreased for BGA and BGT (Table 5).

**Table 5:** Number of different practices that conducted basic geriatric services and the number of postcode areas in which they were located

Basic geriatric Care service		Number			
		2014	2015	2016	2017
Basic geriatric assessment	postcode areas (n)	182	181	179	178
	GP-practice IDs (n)	926	920	900	890
Geriatric treatment	postcode areas (n)	180	181	179	179
	GP-practice IDs (n)	912	903	896	888

**Fig. 1:**
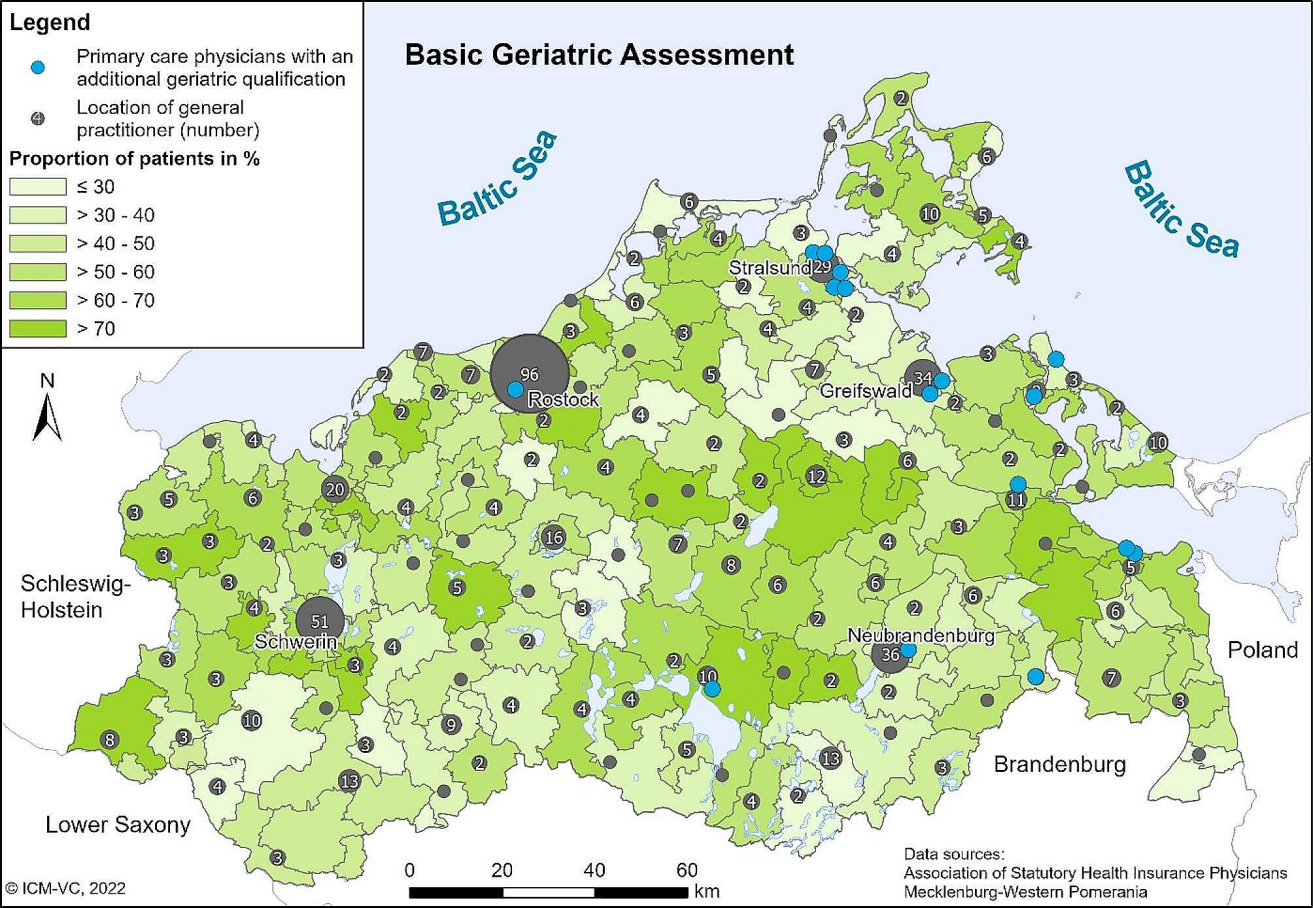
Proportion of eligible patients with at least one BGA of all patients eligible to BGC by postcode areas in 2017. Own presentation

**Fig. 2:**
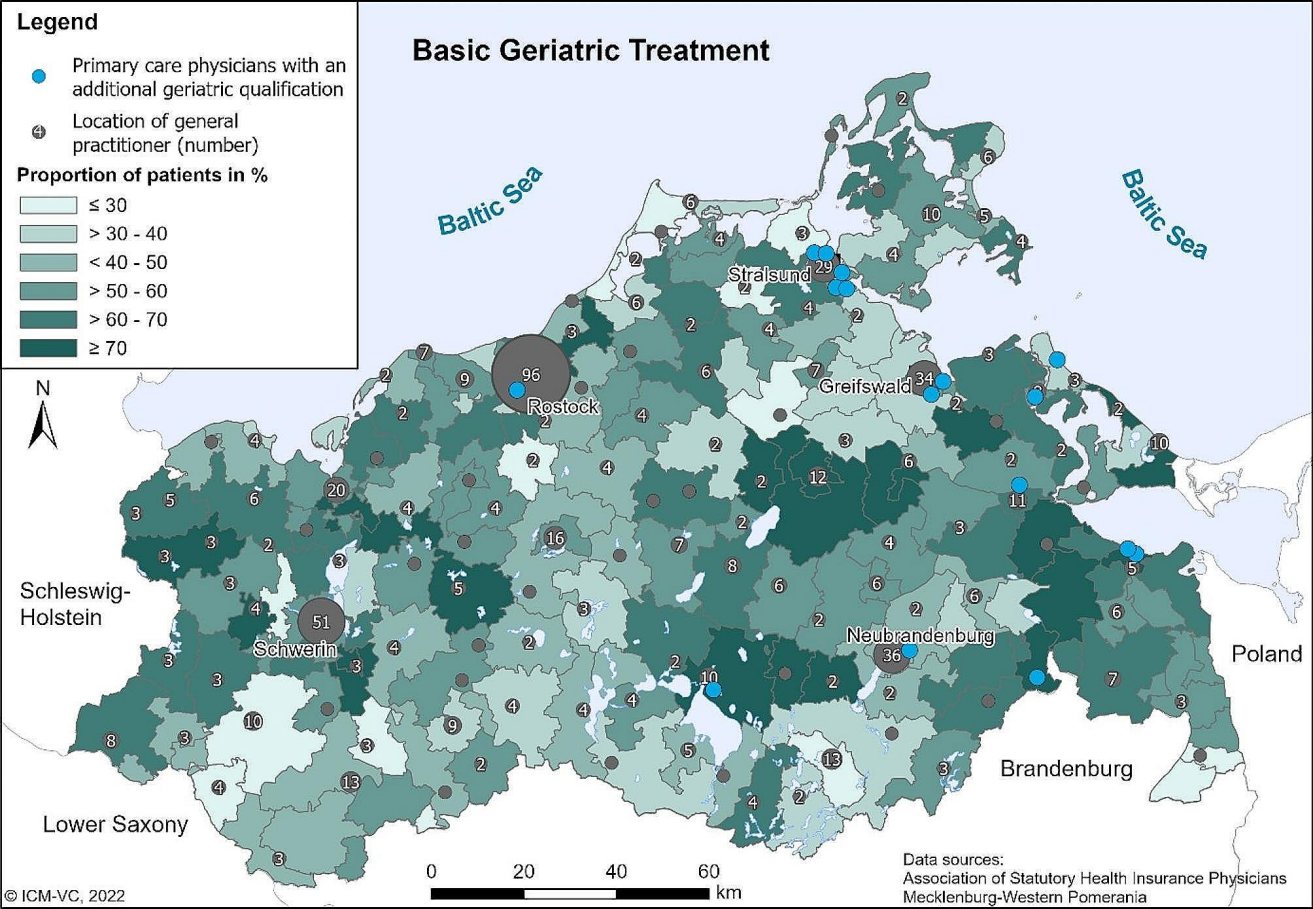
Proportion of eligible patients with at least one BGT of all patients eligible to BGC by postcode areas in 2017. Own presentation

According to the Central Information Register, MWP had a population of 1,631,031 in January 2018, of which 294,503 (18%) were 70 years of age or older. The maps in Figs. 1 and 2 show the spatial distribution of the BGC services in 2017. The maps show the proportion of patients who received at least one BGC service in relation to the total number of eligible patients per postcode area. The maps show a high regional variation in the proportion of treated patients for both BGC services. The proportion of patients who received a BGA ranges from 3.4 to 86.7% across the postcode areas (with 90% of the values between 21.4 and 77.0%). The median is 51.6%. The utilization of BGT ranges from 3.4 to 89.8% (with 90% between 26.9% and 77.1%), the median is 55.4%.

Fewer BGAs were carried out towards the southwest of the Federal State, west of the city of Greifswald and in the southern border region to the Federal State of Brandenburg. More BGAs were carried out towards the centre of the federal state, the east, the north and the northeast (Fig. 1). The map in Fig. 2 shows almost the same picture for BGT with only minor deviations.

Spatial autocorrelations were carried out to analyse the spatial distribution of the BGC services. The calculation of the global Moran’s I resulted in a value of 0.16 (z-score 3.36, p-value 0.0008) of the Moran’s Index for the BGA. The index for the BGT was 0.15 (z-score 3.17, p-value 0.0015). This means that the spatial distribution of both BGC services does not follow a random pattern.

**Fig. 3:**
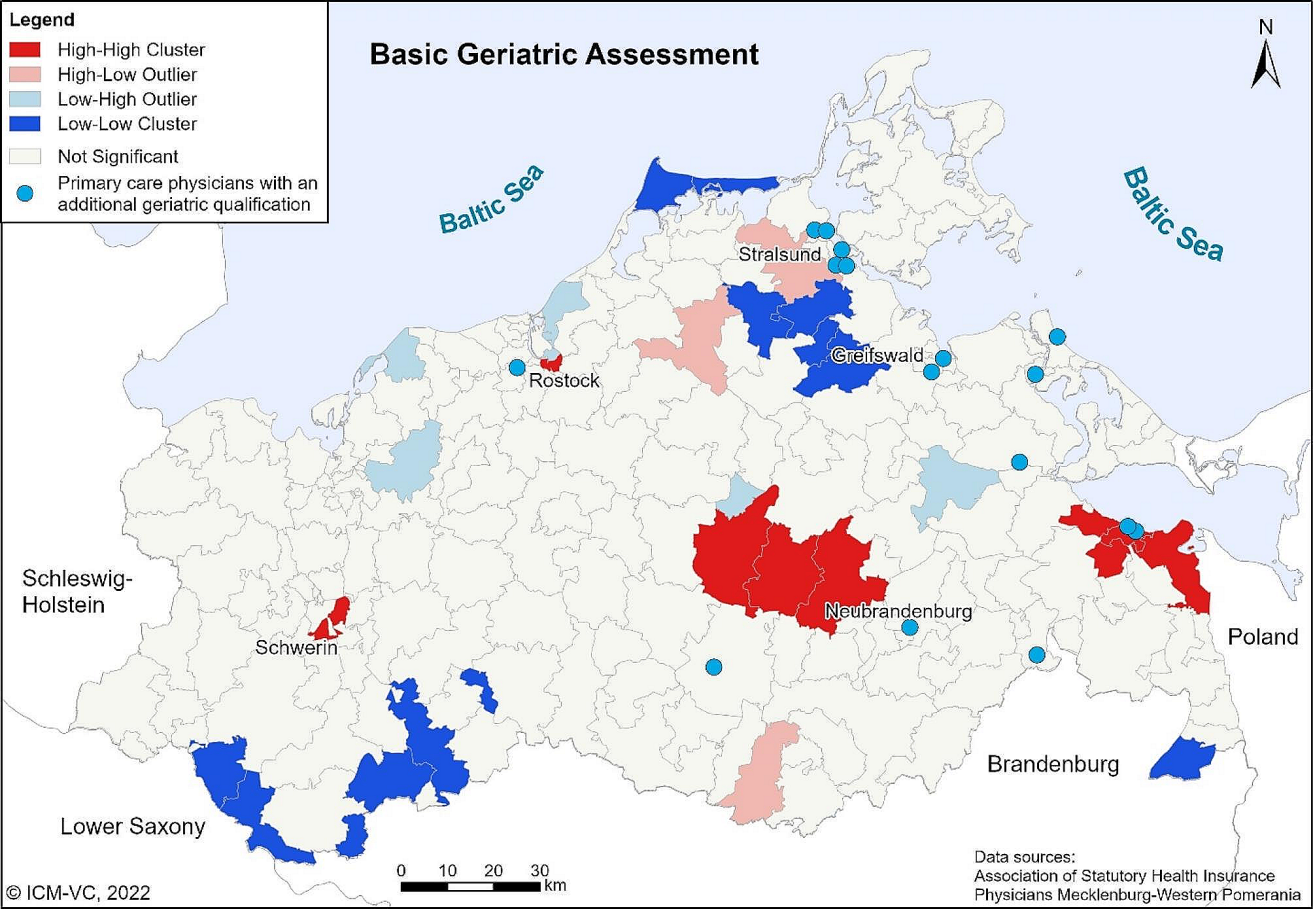
Local Moran’s I for basic geriatric assessment. Own presentation

**Fig. 4:**
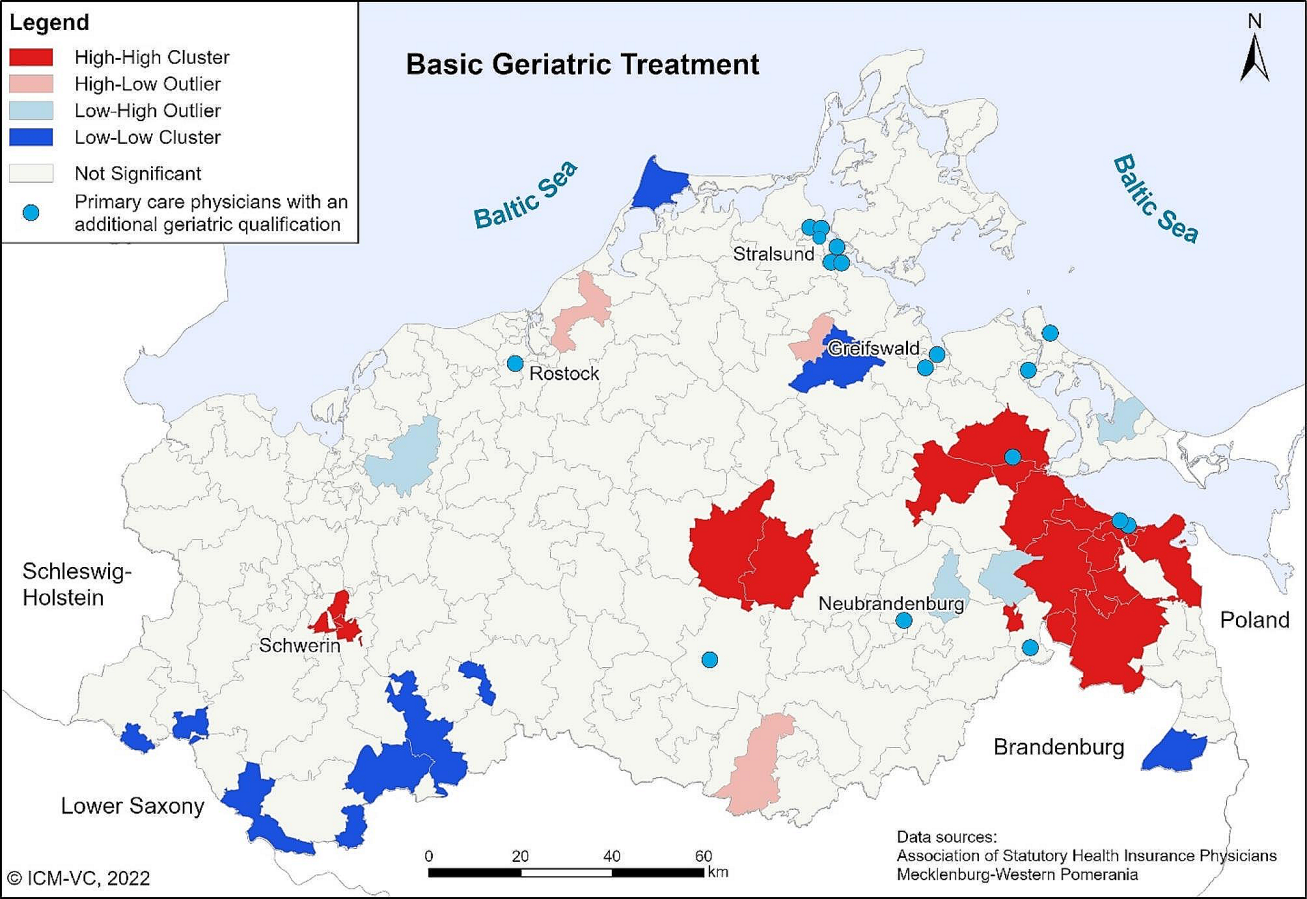
Local Moran’s *I* for basic geriatric treatment. Own presentation

With the Local Moran’s I, regional clusters of high and low utilization of BGC services can be identified. The cartographic visualization of the results of Local Moran’s *I* is shown in Fig. 3 (for BGA) and 4 (for BGT). The light red and light blue postcode areas are outliers. This means that in these areas the rate of treatments performed is very high or very low compared to the neighbouring areas. In the dark red and dark blue areas, high and low rates of BGC are grouped together (clustering). For the BGA, clusters of low utilization can be identified west of Greifswald and at the south border of MWP. Clusters of high utilization can be found in the centre of MWP and in the east at the Polish border (Fig. 3). The results of Local Moran’s I for BGT are similar (Fig. 4).

The spatial evaluation of the SGC services in 2017 showed a more differentiated picture.

The GP consultation as part of the SGC service to determine a possible need for further geriatric treatment in collaboration with an SP was carried out in 27 GP practices in 17 postcode areas. In addition, the specialist part of the consultation with a GP was carried out in 4 practices in 4 postcode areas prior to the CGA. The CGA was actually provided by 3 practices in 3 postcode areas. The patients who received this service lived in 25 different postcode areas. The CGT was conducted in 14 GP practices in 11 postcode areas. Patients who received all 4 SGC services received them in 2 different practices located in 2 postcode areas.

The results of the sensitivity analysis showed that neither the age structure of the regional population nor the density of the GPs in a postcode area are significantly associated with the distribution of the utilization of BGC services.

## Discussion

Between 2014 and 2017, the total number of outpatient geriatric services in MWP remained almost constant. Another nationwide evaluation of the utilization of SGC services showed a stagnation or even reduction in provided SGC services between 2016 and 2020 [26]. At the same time, the proportion of elderly people (> 70 years) in the total population in MWP decreased slightly (because of the birth break after World War II), while the population of very old people (> 80 years) increased [32]. In 27.9% of the postcode regions (*n* = 50), the proportion of eligible geriatric patients who actually received a BGA is < 40%. The number of practice locations, which provided BGC and SGC services, decreased during the observation period.

The results of the Moran’s I analyses show distinct clusters of regions with particularly high or low levels of utilization, respectively. According to the results of the sensitivity analysis, local differences in the density of SHI accredited physicians or age structure were not responsible for the regional disparities of the utilization of BGC.

Considering SGC, regional differences in the utilization are even greater than for BGC. In most of the postcode areas, no patients received SGC. Similarly, the overall utilization of SGC services per year increased just barely (considering the different accounting periods for 2016 and 2017) during the observation period. However, most patients eligible for SGC received BGC services. The proportion of patients who actually received SGC services was very low. Moreover, in 2017, only 12 outpatient practitioners had the qualification to provide a CGA, and only 4 of them actually conducted at least one of the SGC services during the observation period. Patients who received all SGC services (starting with consultation between GP and SP in the forefield, through CGA by a SP, and CGT based on the results of the CGA) were, without exception, consistently treated by one and the same SP practice.

According to a German guideline for geriatric assessments in GP practices the geriatric assessment in GP practices should be primarily used to identify patients with frailty or pre-frailty syndrome and provide adequate care for this vulnerable group [33]. The budget for geriatric services provided to patients of the SHI is limited per practice and year. Services provided beyond this fixed budget are only remunerated with a discount and the provision of further services is usually not economical [34]. Therefore, the GP has to decide which patients are likely to have the highest need for geriatric care [33]. Guidelines recommend a standardized initial screening to identify geriatric patients with a high risk, e.g., for frailty or for chronical conditions, but this is not mandatory. Without such screening, however, the decision of the GP could be biased and some of the patients with an urgent need for geriatric care may not be identified [33].

Different assessment instruments or batteries meet the criteria of the German reimbursement scheme, EBM, for a first BGA to check for the different dimensions of geriatric impairments (cognition, emotion, mobility, etc.). The focus of a BGA is to identify patient-individual needs to prepare an individual care plan, which fosters close communication and joint decision-making to set optimal priorities in therapy [33]. Available assessment instruments differ in terms of their required time and effort and the associated costs.GP might consider some of them as too expensive or time-consuming [33, 35]. Therefore, and because of the limited reimbursement budget, individual practices may provide BGA to a varying extent. This is supported by an online survey among 161 GPs from Lower Saxony in 2012. Due to time constraints geriatric assessments were rarely used despite the fact that most of the practitioners perceived it as useful and effective in order to improve the treatment of geriatric patients. Furthermore, the used instruments did not always match the recommendations of the relevant guidelines [36]. The care of older patients may then take place without geriatric assessments and may be restricted to the treatment of individual diseases without considering functional impairments and resources of the patients. In addition to the guideline for the BGA in GP practices, geriatric care follows a variety of different evidence-based guidelines for the treatment of individual diseases. Without the holistic view that a geriatric assessment conveys, they can add up to excessive or even harmful therapies in the interaction [37].

The very small number of patients receiving SGC could be an indication that older patients may not always be adequately treated in MWP. Obviously, in some regions, the absence of SGC is substituted, e.g., by BGC or inpatient care [38]. However, a controlled trial on frail patients in the age of 65 years or older comparing geriatric and usual care in inpatient as well as in outpatient settings showed for the geriatric care group significant reductions in functional decline and improvements in mental health as well as in quality of life, with no increase in total costs [21].

In a survey of 1,545 healthcare providers of in- and outpatient geriatric health care in Mecklenburg-Western Pomerania and Lower Saxony 71% of the respondents were concerned about the timely availability of SGC for their patients [39]. SGC is low-threshold, better accessible for the patients, and is supposed to be more cost-effective compared to inpatient care [30]. Especially for the CGA, studies yielded improved outcomes in older persons including mental health, functional status, and a trend to longer survival at no increased cost [21, 25, 40, 41]. However, according to our results SGC were provided in very few regions and the regional cooperation between GPs and SPs seems to be low.

A possible reason for the low dissemination of SP practices may be that, although more possibilities for the reimbursement of specialized geriatric care exist since the second half of 2016, the incentives for SP are still considered to be too low compared to the effort needed to acquire the required qualifications [42]. Outpatient physicians have to complete most of the training in inpatient healthcare facilities, which is not always attractive for GP already working in their own practices [39, 43]. Moreover, while geriatrics is acknowledged either as a medical specialty or as a subspecialty in most European countries [44], geriatrics in MWP is only acknowledged as additional qualification [10, 45].

A result of the Moran’s I analysis was the identification of statistically significant clusters with a high utilization of both the BGA and the BGT in the West, in the centre and in the east of MWP. Especially in those regions, geriatric specialized outpatient facilities or healthcare networks with a specific geriatric healthcare concept are located. In the city Schwerin (west cluster), there is a specialized outpatient walk-in-clinic for geriatric patients. In the centre of MWP, a SP provides an innovative outpatient care programme for geriatric patients. The region of Ueckermünde (east cluster) contains a healthcare network that provides SGC and managed care. Thus, we concluded that the presence of active SGC or integrated outpatient geriatric healthcare providers radiate into the region, with the consequence that GP in those regions may be more likely to be informed about the benefits of a comprehensive approach to geriatric patients and also about the reimbursement possibilities of geriatric services. Low utilization clusters can have several causes, from financial incentives to working conditions to the physicians’ qualification. Further research is needed to investigate possible reasons for regionally low utilization rates.

This study has several limitations. The dataset only contains information on the use of outpatient services, although SGC care is also offered by inpatient healthcare providers. Patients may appear in the data several times in one quarter for the same treatment, because he or she changed his or her health insurance company, or switched to another GP practice in that quarter.

In the ASHIP-MWP dataset different types of geriatric assessments have identical reimbursement codes, regardless of whether they are used for screening purposes, to identify patients’ health dimensions needed to be addressed, to monitor the effects of a therapy, or to further clarify impairments [46]. Another limitation is that it contains only patient data of the SHI. However, in 2019, only about 12% of all German inhabitants were not covered by the SHI [30]. In the Federal State of MWP, about 95% of the patients are insured in a SHI.

Various analyses have shown that the utilization of GP increases with age. On average, 80% of the patients ≥ 70 years visit the GP at least once a year [47, 48]. According to a study by Stentzel et al. [49], this proportion rises to almost 100% in the population of people ≥ 85 years in the region of Western Pomerania. Therefore, it can be assumed that the present study represents most of the older inhabitants of MWP and that the risk of bias due to patients without GP contact during the observation period is probably low.

Another limitation is, that the definition for the eligibility of patients to utilize geriatric services used by the ASHIP-MWP is very broad and does not imply actual geriatric needs.

Furthermore, the data contains only those diagnoses and symptoms of a patient that have actually led to a treatment in a GP practice [50]. Moreover, the ASHIP data contains no information on prescribed medication and nursing care services, which could be used to ascertain the severity of a case, or to describe the actual medical situation of a patient. Because of that, the data are not suitable for determining the actual geriatric needs of patients. The geriatric needs of the patients can only be approximately indicated using the proxy variables age, nursing care level and ICD-10 codes indicating geriatric syndromes (Table 1). Therefore, further analyses based on more recent and more comprehensive data sets, like those of the SHI, are needed in order assess whether the provision of BCG and SGC services is based on the patients’ medical needs or not. Moreover, the consideration of such data sources with more medical details would allow a longitudinal comparison of the effectiveness of BGC and SGC services.

Additionally, the ASHIP-MWP data refers to postcode areas, but a few postcode areas cross the border of MWP. While the population figures for all postcode areas are given in full, only the patient numbers from MWP are known for the cross-border postcode areas. The calculated utilization rates can therefore underestimate the actual utilization rates in the border areas.

The availability of GP practices in neighbouring federal states near the border to MWP can also lead to a distortion of the spatial analysis of the distribution of geriatric services in border regions, because inhabitants from MWP may seek care in neighbouring federal states which is not represented in the data.

However, an advantage is that the ASHIP-MWP data include patients from all SHI companies, representing 95% of the population in MWP. The analysis shows to what extent the majority of the older population is provided with BGC and SGC services on a regional level. The ASHIP-MWP reimbursement data had a high level of completeness and, were therefore, a good basis for analyses of the utilization of healthcare services and regional disparities.

## Conclusions

The population-based spatial analysis of geriatric services enable the identification of regional disparities in geriatric care. To the best of our knowledge, no study has analysed the spatial distribution of the use of outpatient geriatric services for a comparatively large population in Germany. This study shows marked differences between regions and between BGC and SGC utilization by in MWP. In clusters of particularly high utilization rates local SP or specialized geriatric care networks seem to foster regional geriatric healthcare. However, more research is needed, including data from all healthcare sectors and health professionals involved in geriatric care with more detailed medical information, in order to comprehensively describe the cross-organizational care of geriatric patients and to compare the effectiveness of BGC and SGC.

Prospectively, a decreasing number of GP will have to treat an increasing number of geriatric patients. Comprehensive, data-based regional planning is a prerequisite for an efficient use of the resources of the healthcare system and the provision of a patient-centered high-quality care that facilitates a healthy ageing in-place system.

### Electronic supplementary material

Below is the link to the electronic supplementary material.

Supplementary Material 1

Supplementary Material 2

## Data Availability

The datasets used and/or analysed during the current study are available from the corresponding author on reasonable request.

## Abbreviations

ASHIP-MWP: Association of Statutory Health Insurance Physicians in Mecklenburg-Western Pomerania
BCG: Basic Geriatric Care
BGA: Basic Geriatric Assessment
BGT: Basic Geriatric Treatment
CGA: Comprehensive Geriatric Assessment
CGT: Comprehensive Geriatric Treatment
EBM: German Abbreviation for the reimbursement catalogue for the Statuary Health Insurance accredited physicians
GP: General Practitioner
MWP: Mecklenburg-Western Pomerania
SGC: Specialized Geriatric Care
SHI: Statutory Health Insurance
SP: Specialized Practitioner

## References

[1] Sander M, Oxlund B, Jespersen A, Krasnik A, Mortensen EL, Westendorp RGJ (2014). The challenges of human population ageing. Age Ageing.

[2] Goins RT, Williams KA, Carter MW, Spencer M, Solovieva T (2005). Perceived barriers to health care access among rural older adults: a qualitative study. JRural Health.

[3] Smith ML, Dickerson JB, Wendel ML, Ahn S, Pulczinski JC, Drake KN (2013). The Utility of Rural and Underserved designations in Geospatial assessments of Distance traveled to Healthcare Services: Implications for Public Health Research and Practice. J Environ Public Health.

[4] Rosenthal TC, Fox C (2000). Access to Health Care for the Rural Elderly. JAMA.

[5] Hämel K, Ewers M, Schaeffer D (2013). Versorgungsgestaltung Angesichts Regionaler Unterschiede. Zeitschrift für Gerontologie Und Geriatrie.

[6] Chan L, Hart LG, Goodman DC (2006). Geographic Access to Health Care for Rural Medicare beneficiaries. J Rural Health.

[7] Thyrian JR, Hertel J, Wucherer D, Eichler T, Michalowsky B, Dreier-Wolfgramm A (2017). Effectiveness and Safety of Dementia Care Management in Primary Care: a Randomized Clinical Trial. JAMA Psychiatry.

[8] Schmader KE, Hanlon JT, Pieper CF, Sloane R, Ruby CM, Twersky J (2004). Effects of geriatric evaluation and management on adverse drug reactions and suboptimal prescribing in the frail elderly. Am J Med.

[9] Borchelt MK, Lübke G. N Abgrenzungskriterien Der Geriatrie. In: e.V. BdK-GE, editor. Berlin2004.

[10] Deutsche Gesellschaft für Geriatrie. Was ist Geriatrie?. 2018. Available from: http://www.dggeriatrie.de/nachwuchs/91-was-ist-geriatrie.html.

[11] Lacas A, Rockwood K (2012). Frailty in primary care: a review of its conceptualization and implications for practice. BMC Med.

[12] Fried LP, Ferrucci L, Darer J, Williamson JD, Anderson G. Untangling the concepts of disability, frailty, and comorbidity: implications for improved targeting and care. The journals of Gerontology Series A: Biological sciences and Medical sciences. 2004; 59(3):M255–63; 10.1093/gerona/59.3.m255. 10.1093/gerona/59.3.m255 15031310

[13] Buttery AK, Busch MA, Gaertner B, Scheidt-Nave C, Fuchs J (2015). Prevalence and correlates of frailty among older adults: findings from the German health interview and examination survey. BMC Geriatr.

[14] Robert Koch Institute. DEGS1. (2008–2011) 2015 [updated 09.04.2015]. Available from: https://www.rki.de/DE/Content/Gesundheitsmonitoring/Studien/Degs/degs_w1/degs_w1_node.html;jsessionid=A5C4FEE340EE5EF05E6DB1EEEEEB40AD.internet092.

[15] Vassallo M, Poynter L, Kwan J, Sharma JC, Allen SC (2016). A prospective observational study of outcomes from rehabilitation of elderly patients with moderate to severe cognitive impairment. Clin Rehabil.

[16] Tarazona-Santabalbina FJ, Belenguer‐Varea Á, Rovira Daudi E, Salcedo Mahiques E, Cuesta Peredó D, Doménech‐Pascual JR (2015). Severity of cognitive impairment as a prognostic factor for mortality and functional recovery of geriatric patients with hip fracture. Geriatr Gerontol Int.

[17] Hajek A, Brettschneider C, Van den Bussche H, Kaduszkiewicz H, Oey A, Wiese B (2018). Longitudinal analysis of outpatient physician visits in the oldest old: results of the AgeQualiDe prospective cohort study. J Nutr Health Aging.

[18] Michalowsky B, Thyrian JR, Eichler T, Hertel J, Wucherer D, Flessa S (2016). Economic analysis of formal care, informal care, and productivity losses in primary care patients who screened positive for dementia in Germany. J Alzheimers Dis.

[19] Multimorbidity AGSEPotCoOAw (2012). Patient-centered care for older adults with multiple chronic conditions: a Stepwise Approach from the American Geriatrics Society. J Am Geriatr Soc.

[20] National Association of Statutory Health Insurance Physicians (abbreviated in German as KBV). Vereinbarung von Qualitätssicherungsmaßnahmen nach § 135 Abs. 2 SGB V zur spezialisierten geriatrischen Diagnostik (Inkrafttreten: 01.07.2016). 2016. [updated 26.01.2022]. Available from: https://www.kbv.de/html/geriatrie.php.

[21] Cohen HJ, Feussner JR, Weinberger M, Carnes M, Hamdy RC, Hsieh F (2002). A controlled trial of inpatient and outpatient geriatric evaluation and management. N Engl J Med.

[22] Applegate WB, Miller ST, Graney MJ, Elam JT, Burns R, Akins DE (1990). A randomized, controlled trial of a geriatric assessment unit in a community rehabilitation hospital. N Engl J Med.

[23] Morley JE (2017). Rapid Geriatric Assessment: secondary Prevention to Stop Age-Associated disability. Clin Geriatr Med.

[24] Mazya AL, Garvin P, Ekdahl AW (2019). Outpatient comprehensive geriatric assessment: effects on frailty and mortality in old people with multimorbidity and high health care utilization. Aging Clin Exp Res.

[25] Romera-Liebana L, Orfila F, Segura JM, Real J, Fabra ML, Möller M (2018). Effects of a primary care-based multifactorial intervention on physical and cognitive function in Frail, Elderly individuals: a Randomized Controlled Trial. J Gerontol Biol Sci Med Sci.

[26] National Association of Statutory Health Insurance Physicians (abbreviated in German as KBV). Geriatrie - Evaluation 2016 [updated 26.01.2022]. Available from: https://www.kbv.de/html/geriatrie.php.

[27] Kolb G, Breuninger K, Gronemeyer S, van den Heuvel D, Lübke N, Lüttje D (2014). 10 Jahre geriatrische frührehabilitative Komplexbehandlung Im DRG-System. Zeitschrift für Gerontologie Und Geriatrie.

[28] Steinhagen-Thiessen E, Hamel G, Lüttje D, Oster P, Plate A, Vogel W. Geriatrie—quo vadis? Zeitschrift für Gerontologie undGeriatrie. 2003; 36(5):366–77; 10.1007/s00391-003-0162-5. 10.1007/s00391-003-0162-5 14579064

[29] Plate A, Armbruster S, Meinck M, editors. Neue Wege in der ambulanten Versorgung: Geriatrische Schwerpunktpraxis (GSP) und Geriatrische Institutsambulanz (GIA) als Innovator einer spezialisierten Versorgung multimorbider Menschen im höheren Lebensalter? Gesundheit, Alter, Pflege, Rehabilitation-Recht und Praxis im interdisziplinären Dialog; 2017: Nomos Verlagsgesellschaft mbH & Co. KG; ISBN: 3848734354.

[30] Verband der Ersatzkassen (vdek). Daten zum Gesundheitswesen: Versicherte 2020 [updated 20.04.2021]. Available from: https://www.vdek.com/presse/daten/b_versicherte.html.

[31] Einheitlicher Bewertungsmaßstab (EBM). (Enacted with effect from 01/06/2020 considering the current decisions until finally the 508th session of the Bewertungsausschuss, the 66th session of the Erweiterter Bewertungsausschuss and the 53th session of the ergänzter Bewertungsausschuss, 2020).

[32] 13. koordinierte Bevölkerungsvorausberechnung nach Bundesländern 2015 [Internet], Statistisches Bundesamt. 2015 [cited 07 Jan 2019]. Available from: https://service.destatis.de/laenderpyramiden/.

[33] Bergert F, Braun M, Feßler J, Hüttner U, Kluthe B, Popert U et al. Hausärztliche Leitlinie. Geriatrisches Assessment in der Hausarztpraxis. Deutsche Gesellschaft für Allgemeinmedizin und Familienmedizin (DEGAM), Berlin; Arbeitsgemeinschaft der Wissenschaftlichen Medizinischen Fachgesellschaften, AWMF. 2017.

[34] National Association of Statutory Health Insurance Physicians (abbreviated in German as KBV). Honorar - Honorarverteilung und -berechnung. 2021; Available from: https://www.kbv.de/html/1019.php.

[35] Junius-Walker U, Daether-Kracke N, Krause O (2016). It‘s MAGIC–einfaches geriatrisches Basisassessment für die Hausarztpraxis validiert. Z für Allgemeinmedizin.

[36] Theile G, Winter A, Hummers-Pradier E, Junius-Walker U (2012). Das Geriatrische Basisassessment in Der Hausarztpraxis. Zeitschrift für Gerontologie Und Geriatrie.

[37] Wehling M (2011). Guideline-driven polypharmacy in elderly, multimorbid patients is basically flawed: there are almost no guidelines for these patients. J Am Geriatr Soc.

[38] van den Heuvel D, Veer A, Greuel HW (2014). Geriatrische Versorgungsstrukturen in Deutschland. Zeitschrift für Gerontologie Und Geriatrie.

[39] Knorr M, Beyer A, Radicke F, Thomé-Soós F, Hoffmann W, van den Berg N (2020). Geriatrische Versorgung in ländlichen Regionen: Ergebnisse Aus Zwei standardisierten Befragungen Von Leistungserbringern Und Akteuren. Zeitschrift für Evidenz Fortbildung Und Qualität Im Gesundheitswesen.

[40] Boult C, Boult LB, Morishita L, Dowd B, Kane RL, Urdangarin CF (2001). A randomized clinical trial of outpatient geriatric evaluation and management. J Am Geriatr Soc.

[41] Burns R, Nichols LO, Martindale-Adams J, Graney MJ (2000). Interdisciplinary geriatric primary care evaluation and management: two-year outcomes. J Am Geriatr Soc.

[42] Kolb G (2017). Geriatrie Oder Geriatrisierung Der Medizin. Zeitschrift für Gerontologie Und Geriatrie.

[43] Swoboda W, Hermens T (2011). Geriatrie in Der Ambulanten Versorgung durch hausärztlich tätige Internisten. Der Internist.

[44] Pitkälä KH, Martin F, Maggi S, Jyväkorpi S, Strandberg T (2018). Status of geriatrics in 22 countries. J Nutr Health Aging.

[45] Deutsche Gesellschaft für Geriatrie. Verzeichnis der Weiterbildungsberechtigten. 2020 Available from: https://www.dggeriatrie.de/aus-und-weiterbildung/verzeichnis-weiterbildungsberechtigte#/.

[46] Krupp S, Frohnhofen H. S1-LeitlinieGeriatrisches Assessment der Stufe 2. 2019; Available from: https://www.awmf.org/leitlinien/detail/ll/084-002.html.

[47] Krause L, Dini L, Prütz F (2020). Inanspruchnahme gynäkologischer und allgemeinärztlicher Leistungen Durch Frauen ab 50 Jahren. J Health Monit.

[48] Rattay P, Butschalowsky H, Rommel A, Prütz F, Jordan S, Nowossadeck E (2013). ­­­Inanspruchnahme Der ambulanten und stationären medizinischen Versorgung in Deutschland. Bundesgesundheitsblatt - Gesundheitsforschung - Gesundheitsschutz.

[49] Stentzel U, Bahr J, Fredrich D, Piegsa J, Hoffmann W, van den Berg N (2018). Is there an association between spatial accessibility of outpatient care and utilization? Analysis of gynecological and general care. BMC Health Serv Res.

[50] Hoffmann W, Bobrowski C, Fendrich K (2008). Sekundärdatenanalyse in Der Versorgungsepidemiologie. Bundesgesundheitsblatt-Gesundheitsforschung-Gesundheitsschutz.

