# Supplementary material for: Outpatient geriatric health care in the German federal state of Mecklenburg-Western Pomerania: a population-based spatial analysis of claims data
Source: BMC Health Serv Res. 2024 Apr 12;24:458. doi: 10.1186/s12913-024-10888-2 (PMC11010346; doi:10.1186/s12913-024-10888-2)
Supplement: Supplementary file 1 — Supplementary Material 1 [file 12913_2024_10888_MOESM1_ESM.pdf]

Table A1: Synoptic overview about the most important key facts of basic geriatric care (BGC) and specialized geriatric care (SGC) in Germany and Mecklenburg-Western Pomerania (MWP).

	**Basic Geriatric Care (BGC)**	**Specialized Geriatric Care (SGC)**
**Comprised Services**	Basic Geriatric Assessment (BGA)<br>Basic Geriatric Treatment (BGT)	Comprehensive Geriatric Assessment (CGA)<br>Comprehensive Geriatric Treatment (CGT)
**Addressed Patients**	• Age ≥ 70 and one geriatric syndrome (see table 1) or an approved care level according to the statutory long-term insurance<br>• Patients at any age diagnosed with Alzheimer disease, Dementia, or Parkinson	• Age ≥ 70 and two geriatric syndrome (see table 1) or one geriatric syndrome and an approved care level according to the statutory long-term insurance
**Authorized healthcare providers**	• General practitioners (GP)	• Specialized geriatric practitioners (SP)<br>• General practitioners (GP) with an additional qualification in geriatrics<br>• Geriatric inpatient ambulances (GIA) or hospital doctors authorized for taking part in the outpatient geriatric care by the Association of Statuary Health Insurance Physicians (ASHIP)
**Content**	• Standardized Assessment and Monitoring of several health dimensions of the patient (e.g., cognition, mobility, social situation, etc.)<br>• Prescription, coordination and management of therapies<br>• The GP monitors the therapy progress and the patients' medications	• Comprehensive standardized Assessment of the health and social situation of the patient<br>• Team-based approach towards the definition and monitoring of treatment goals and a treatment plan, including frequent case conferences of the SP and other involved physicians, therapists or caregivers.
**Reimbursement Codes**	GOP 03360: BGA<br>GOP 03362: BGT<br>GOP 30980: Consultation with SP in order to determine the need for a CGA<br>GOP 30988: SGT based on the results of a CGA	GOP 30981: Consultation with GP in order to determine the need for a CGA<br>GOP 30984-30986: CGA and additional charge if a more extensive CGA is needed<br>GOP 30988: SGT based on the results of a CGA<br>GOP 01321: Fixed rate for GIA
**Eligible Patients in MWP in 2017**	221,654 patients (75.3% of all inhabitants of Mecklenburg-Western Pomerania aged ≥70 years)	95,171 patients (32.3% of the population of Mecklenburg-Western Pomerania ≥70 years
**Prevalence MWP in 2017**	BGA: 113,581 patients (38,6 % of the inhabitants aged 70 years or older in MWP)<br>BGT: 118,755 patients (40,3 % of the inhabitants aged 70 years or older in MWP)	CGA: 129 patients (0,04 % of the inhabitants aged 70 years or older in MWP)<br>CGT: 89 patients (0,03 % of the inhabitants aged 70 years or older in MWP)
