# Supplementary material for: Outpatient geriatric health care in the German federal state of Mecklenburg-Western Pomerania: a population-based spatial analysis of claims data
Source: BMC Health Serv Res. 2024 Apr 12;24:458. doi: 10.1186/s12913-024-10888-2 (PMC11010346; doi:10.1186/s12913-024-10888-2)
Supplement: Supplementary file 2 — Supplementary Material 2 [file 12913_2024_10888_MOESM2_ESM.pdf]

Outpatient Geriatric Health Care in Germany – Reimbursement schemes

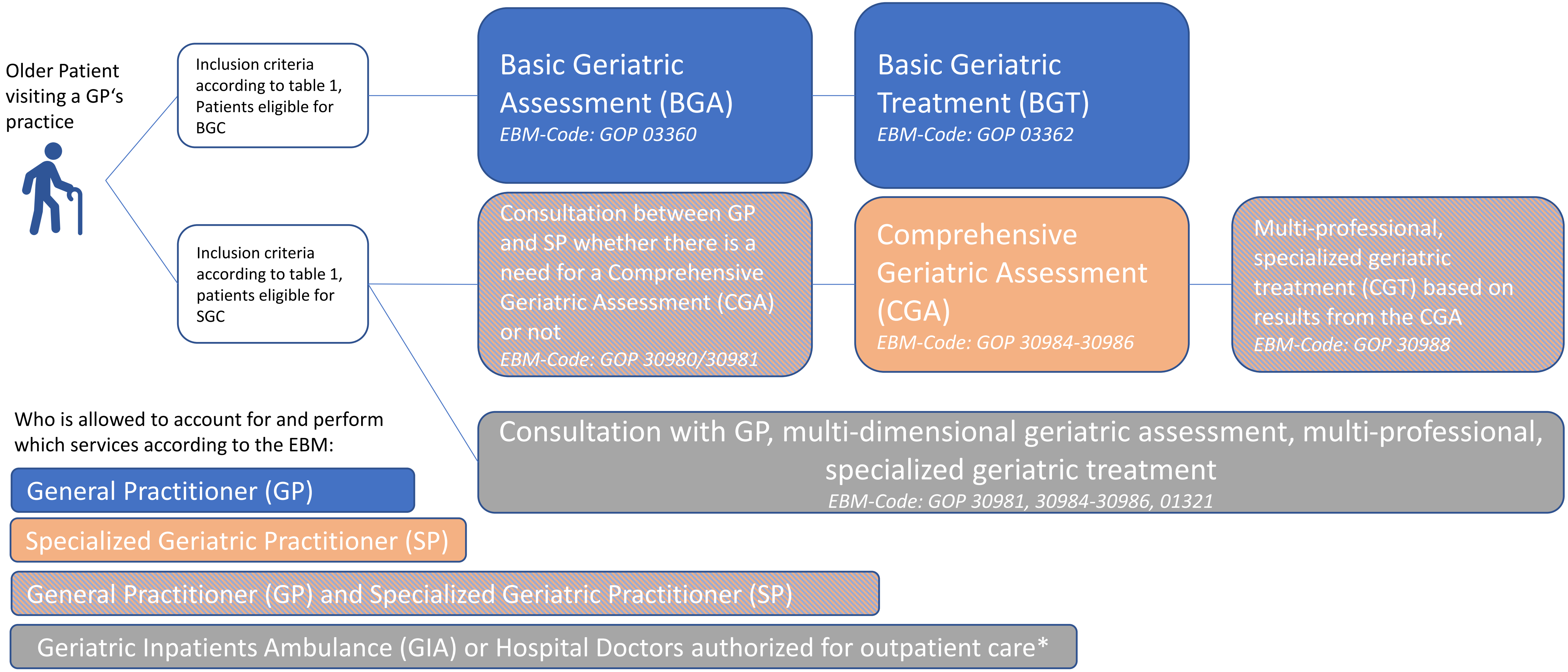

Figure A2: Definitions of geriatric patients applicated by the ASHIP-MWP according to the national ASHIP reimbursement catalogue (EBM) that determines which services can be provided to which kind of geriatric patient. BGC – Basic Geriatric Care, SGC – Specialized Geriatric Care.

*Not existing in Mecklenburg-Western Pomerania
